# Intestinal Bacteria Composition and Translocation of Bacteria in Inflammatory Bowel Disease

**DOI:** 10.1371/journal.pone.0170034

**Published:** 2017-01-18

**Authors:** Spyros Vrakas, Konstantinos C. Mountzouris, George Michalopoulos, George Karamanolis, George Papatheodoridis, Charalampos Tzathas, Maria Gazouli

**Affiliations:** 1 Gastroenterology Department, Tzaneion General Hospital, Piraeus, Greece; 2 Department of Nutritional Physiology and Feeding, Agricultural University of Athens, Athens, Greece; 3 Gastroenterology Unit, 2nd University Surgical Department Medical School, National and Kapodistrian University of Athens, Athens, Greece; 4 Department of Gastroenterology, Medical School, National and Kapodistrian University of Athens Laiko Hospital of Athens, Athens, Greece; 5 Department of Basic Medical Sciences, Laboratory of Biology, Medical School, National and Kapodistrian University of Athens, Athens, Greece; Universita degli Studi di Sassari, ITALY

## Abstract

**Background:**

Live commensal intestinal bacteria are present in the peripheral blood where they can induce inflammation.

**Objective:**

To evaluate the intestinal bacteria composition and translocation of bacteria in IBD.

**Methods:**

Both blood and tissue biopsy samples were collected from adult patients with active/inactive Crohn’s disease (CD), active/inactive ulcerative colitis (UC) and healthy individuals. Most of the patients were newly diagnosed and none of them received antibiotics. Using a reverse transcription–quantitative real-time PCR (RT-qPCR) method, we determined the composition of microbiota. *NOD2/CARD15* genotyping was also studied.

**Results:**

Total bacterial DNA concentration was increased in tissue and blood samples of IBD patients compared to healthy controls. Furthermore, the active IBD cases had higher total bacterial DNA concentration levels compared to the inactive cases. Three species characterized dysbiosis in IBD, namely an increase of *Bacteroides spp* in active and inactive IBD samples, and a decrease in *Clostridium leptum* group (IV), and *Faecalibacterium prausnitzi* in both active and inactive IBD patients. No significant association between bacterial translocation and *NOD2/CARD15* mutations was found.

**Conclusions:**

The composition of the microbiota in IBD patients differs from that of healthy controls. The high rate of bacterial DNA in the blood samples indicates translocation in inflammatory bowel disease.

## Introduction

The exact cause of Inflammatory bowel disease (IBD), consisting of Crohn’s disease (CD) and ulcerative colitis (UC) is unknown. However, IBD is thought to be driven by an abnormal immune response to intestinal microbiota in genetically predisposed individuals[1]. The intestinal microbiota is essential for the host energy balance, immune regulation and homeostasis, as well as for the host metabolism such as the breakdown of complex dietary carbohydrates, the mucus, and the production of organic acids to maintain an appropriate pH environment in the gut [2]. Dysbiosis of the intestinal microbiota has been shown in patients with several gastrointestinal disorders including IBD [3]. Additionally, several studies supported that the gut microbiota of patients with active IBD is depleted, is constituted of smaller number bacterial species, and in more unstable over time compared to those of patients with inactive IBD and healthy individuals [4,5]. Molecular analyses of the gut microbiota have also revealed differences in microbiota composition between CD and UC. Specifically, the gut microbiota in healthy people is dominated by the bacteria phyla *Firmicutes*, and *Bacteroidetes* and to a lesser extent by *Proteobacteria*, and *Verrucomicrobia* [6]. Concerning IBD, Wills et al [7] reported larger changes in the bacterial community composition between remission and exacerbation in CD patients in comparison to UC patients. Hansen et al [8] demonstrated an increase in *Faecalibacterium prausnitzi*, and a reduction in bacterial diversity in newly diagnosed pediatric CD patients but not in UC patients. Microbial changes were identified by biopsying colonic mucosa. Fyderek et al [9] showed a predominance of *Streptococcus spp*. in inflamed mucosa of CD adolescent patients, while *Lactobacillus spp*. were predominant in UC patients. Forbes JD et al [10] also found an increase in the levels of *Bacteroidetes*, and *Fusobacteria* in inflamed CD mucosa, although *Proteobacteria*, and *Firmicutes* were more frequently observed in inflamed UC patients. Gophna et al [11], and Bibiloni et al [12] showed a significant increase of *Bacteroidetes* in CD than in UC patients. However, in an Indian population, Kabeerdoss et al [13] found greater *Bacteroides* and *Lactobacillus* concentration in inflamed colonic mucosa of patients with UC in comparison to CD patients.

Recently Amar et al [14] suggested that in a mice model during high-fat diet-induced diabetes, live commensal intestinal bacteria were translocated in the adipose tissue and the peripheral blood where they could induce inflammation. In addition, Cani et al [15] have demonstrated in a mice model during high-fat diet-induced diabetes that the lipopolysaccharide (LPS) produced by intestinal Gram-negative bacteria, which is a well-recognized pro-inflammatory molecule, that can translocate to the bloodstream from a leaky gut, and causes metabolic endotoxemia, inflammation, and associated disorders. More recently, Sato et al [16] demonstrated gut dysbiosis and possible blood bacterial translocation in patients with type 2 diabetes. Transient translocation of members of the intestinal microbial flora to blood has been supported by an increasing number of reports connecting this finding with a variety of infectious, inflammatory, as well as non-infectious and non-inflammatory diseases [17–20]. Regarding IBD there are only few data in the literature. Gutiérrez et al [21, 22] demonstrated the presence of bacterial DNA in serum samples from IBD patients, and have also shown that proinflammatory cytokines were increased in CD patients with bacterial-DNA in blood and supported that NOD2/CARD15 seems to play a key role in the regulation of this response.

Since this process can trigger inflammation the aim of the present study was to evaluate the intestinal bacterial composition and its translocation to blood in IBD patients in Greece, through a cohort study.

## Materials and Methods

### Study subjects

For this study IBD patients (6 active CD, 6 inactive CD, 14 active UC, 6 inactive UC patients) who regularly visited the Gastroenterology Department, of Tzaneion General Hospital between 2015 and 2016 were recruited, and agreed to participate in the study. Twenty control subjects who visited the Gastroenterology Department, of Tzaneion General Hospital for screening colonoscopy were also recruited. The diagnosis of IBD was based on standard clinical, endoscopic, radiological, histological criteria, and colonoscopy [23]. Patients with the following conditions were excluded from the study: 1) autoimmune, inflammatory, and infectious related diseases, 2) malignancy, and 3) a history of treatment with antibiotics within 3 months of study participation. Most of the patients were newly diagnosed and none of them received antibiotics. Biopsies were collected only from the inflamed sites in active UC and CD. In inactive IBD and healthy patient’s biopsies were taken from five sites (cecum-ascending, tranverse, descending, sigmoid, rectum). Two biopsies were collected from every site using standard biopsy forceps. Blood samples were collected from all the participants. The study protocol was approved by the Hospital Ethics Committee (Hellenic Ministry of Health, 1^st^ Y.PE Attikis scientific council 270/12-03-2015) and written informed consent was obtained from each patient and control before enrollment in the study. Disease activity in UC was defined using the endoscopy Mayo score (Inactive: Mayo score = 0, active: Mayo score = 1–3) and in Crohn’s disease using the SES-CD score (Inactive: SES CD = 0, Active: SES CD≥1).

### Biopsies and blood bacterial composition

DNA was extracted from biopsies and blood samples using the Nucleospin tissue kit (Macherey-Nagel, Düren, Germany) according to the manufacturer’s instructions. For each sample, the purified DNA was eluted in 100 μL elution buffer and the quality and quantity of the preparations were determined by spectrophotometry (NanoDrop-1000, Thermo Fisher Scientific, Waltham, UK) and stored at −20°C. The samples were analyzed for the following selected IBD microbiota constituents: total bacteria, *Escherichia coli*, *Lactobacillus* spp., *Bifidobacterium* spp., *Bacteroides* spp., *Clostridial coccoides* group (XIVa), *Clostridium leptum* group (IV), and *Faecalibacterium prausnitzii*. The primers used are presented in Table 1 [24, 25]. Total bacteria concentration was determined as previously described [25]. Standard curves were constructed from 10-fold serial dilutions of known concentrations of reference strain genomic bacterial DNA and cfu plotted against the respective cycle threshold (Ct) value. Sample DNA microbiota constituents was determined by interpolating the Ct values obtained from the samples into the appropriate standard calibration curve. Group and species-specific 16S rRNA determination was performed as described by Wang et al [24]. To determine the influence of biopsy specimen sizes of mucosal tissue, the human cell numbers were quantified using primers specific for the *GAPDH* gene to determine the total number of mucosa-associated bacteria in the biopsy specimens. To reduce the quantitative error of the detected bacteria and to characterize the changes in bacterial copies, the abundance of 16S rRNA gene copies was calculated from standard curves, and specific bacterial groups were expressed as a percentage of the total bacteria determined by the universal primers. In all cases, real time PCR was performed using an ABI 7500 real time PCR system (Applied Biosystems, CA).

**Table 1:** Primers targeting 16S rRNA gene used for determination of microbiota composition by real time PCR.

Target group or organism	Sequence (5'-3')	Annealing temperature (°C)	References
All bacteria	Forward: ACTCCTACGGGAGGCAGCAG Reverse: ATTACCGCGGCTGCTGG	60	25
*E*. *coli*	Forward: GTTAATACCTTTGCTCATTGA Reverse: ACCAGGGTATCTAATCCTGTT	61	24
*Lactobacillus spp*.	Forward: GCAGCAGTAGGGAATCTTCCA Reverse: GCATTYCACCGCTACACATG	61.5	24
*Bifidobacterium spp*.	Forward: AGGGTTCGATTCTGCTCAG Reverse: CATCCGGCATTACCACCC	62	24
*Bacteroides spp*.	Forward: GTCAGTTGTGAAAGTTTGC Reverse: CAATCGGGAGTTCTTCGTG	61.5	24
*C*. *coccoides group (XIVa)*	Forward: AAATGACGGTACCTGACTAA Reverse: CTTTGAGTTTCATTCTTGCGAA	61	24
*C*. *leptum group (IV)*	Forward: GTTGACAAAACGGAGGAAGG Reverse: GACGGGCGGTGTGTACAA	60	24
*F*. *prausnitzii*	Forward: AGATGGCCTCGCGTCCGA Reverse: CCGAAGACCTTCTTCCTCC	61.5	24
*GAPDH*	Forward: GAA GAT GGT GAT GGG ATT TC Reverse: GAA GGT GAA GGT CGG AGT C	Based on detected bacterial Tm	27

### *NOD2/CARD15* Genotyping

*NOD2/CARD15* (R702W, G908R, and 3020insC) polymorphisms were evaluated as previously described [26].

### Statistical analysis

Comparisons were made using Student’s t test or one-way analysis of variance for variables with normal distributions. For non-normal distributions, the Mann-Whitney U test was used for comparisons between two groups, and the Kruskal-Wallis method was used to compare more than two groups. *P* values of <0.05 were considered statistically significant. Specific bacterial levels were expressed as a percentage of the total bacterial levels of each sample.

## Results

The clinical and analytical characteristics of patients included in the study are presented in Table 2.

**Table 2:** Clinical and analytical characteristics of patients included in the study.

	CD (n = 12)	UC (n = 20)	Controls (n = 20)
Age (years± SD)	36.7±14.7	58±15.9	60.6±10.8
Sex (male/female)	7/5	15/5	11/9
Disease (Active/Inactive)	6/6	14/6	
Disease duration (years)	6.6±7.9	12.8±11.9	
Smoking habits n (%)	7 (58.33%)	6 (30%)	7 (35%)
Montreal A (age of onset) n (%)			
A1 (≤16)	3 (25%)		
A2 (17–40)	5 (41.6%)		
A3 (≥41)	4 (33.3%)		
Montreal L (location) n (%)			
L1 (ileal)	0		
L2 (colonic)	3 (25%)		
L3 (ileocolonic)	9 (75%)		
Montreal B (behavior) n (%)			
B1(nonstricturing, nonpenetrating)	10 (83.33%)		
B2 (stricturing)	0		
B3 (penetrating)	0		
p (perianal disease)	2 (16.67%)		
Montreal			
E1 ulcerative proctitis		2 (10%)	
E2 left sided ulcerative colitis		10 (50%)	
E3 extensive ulcerative colitis		8 (40%)	
SES CD	6.9±10.1		
Endoscopy Mayo Score			
0		6 (30%)	
1		3 (15%)	
2		7 (35%)	
3		4 (20%)	
Therapy n (%)			
5 ASA	6 (50%)	14 (70%)	
5 ASA and Azathioprine	1 (8.3%)	1 (5%)	
5 ASA and Steroids	1 (8.3%)	1 (5%)	
Azathioprine	2 (16.6%)	0	
No treatment	2 (16.6%)	4 (20%)	
CRP (mg/dL)	5.9±8.3	8.2±10.9	

**Fig 1:**
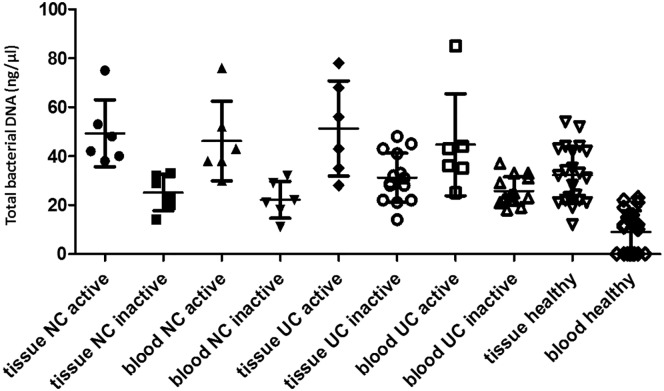
Total bacterial DNA (ng/μl) per patient and healthy controls. Samples were grouped according to their pathological stage: active NC and UC, and inactive NC and UC.

The average of total bacterial quantifications and the comparison in tissue and blood in each group are summarized in Table 3 and Fig 1. All CD and UC patients and 11 out of 20 healthy individuals (55%) presented bacterial DNA in the blood. The total concentrations of bacteria DNA in the tissue and blood samples did not differ significantly between CD and UC patients. However, tissue and blood bacterial DNA concentrations were significantly higher in active CD and UC compared to those from inactive CD and UC, respectively, as well as those from healthy subjects. Furthermore, total bacterial DNA concentration was significantly higher in active cases of CD and UC compared to the inactive CD and UC samples, or to healthy controls (Table 3). No statistical significant differences were observed between CD and UC cases, as well as between inactive cases and healthy controls.

**Table 3:** Total bacteria DNA (ng/μl) in tissue and blood microbiota.

	Total Bacteria DNA (ng/μl) ± SD	*P*
Tissue NC active	49.33 ± 13.74	*vs* Tissue Healthy: 0.02
		*vs* Tissue NC inactive: 0.002
Tissue NC inactive	25.17 ± 7.47	
Blood NC active	46.17 ± 16.30	*vs* Blood Healthy: 0.0002
		*vs* Blood NC inactive: 0.009
Blood NC inactive	22.17 ± 7.57	
Tissue UC active	51.33 ± 19.45	*vs* Tissue Healthy: 0.03
		*vs* Tissue UC inactive: 0.032
Tissue UC inactive	31.21 ± 10.02	
Blood UC active	44.67 ± 20.91	*vs* Blood Healthy: 0.0002
		*vs* Blood UC inactive: 0.005
Blood UC inactive	25.71 ± 5.85	
Tissue Healthy	32.29 ± 11.57	
Blood Healthy	9.00 ± 8.67	

**Fig 2:**
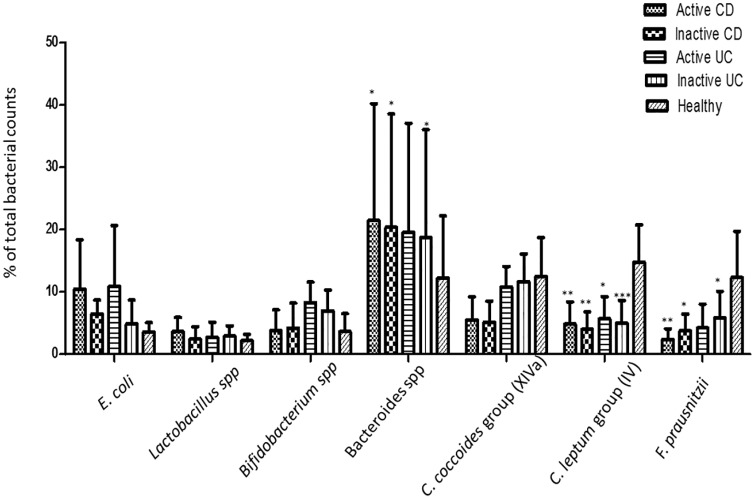
Quantification of dominant bacterial in biopsies. The bars represent the percentage of total bacterial counts in active and inactive CD, in active and inactive UC cases and in heathy samples. * *P* < 0.05; ** P < 0.01; *** *P* < 0.001.

Regarding the dominant bacteria found in IBD patients and healthy controls biopsies, the average bacterial quantifications of biopsies, in biopsied cases from each group are illustrated in Table 4 and in Fig 2. The proportions of *E*. *coli* were found to be numerically higher in active CD and UC but without a significant difference compared to healthy controls. The proportions of *Lactobacillus* spp and *Bifidobacterium* spp were low in CD and UC patients and comparable to those in healthy controls. In contrast, the proportions of *Bacteroides* spp were higher in active CD, as well as in inactive CD and UC than in the controls (*P* < 0.05); they were higher in active UC samples than in controls too, but without reaching statistical significance. Regarding the proportions of *C*. *coccoides* group (XIVa), they were numerically lower in active CD and UC patients compared to patients with inactive CD and UC or healthy controls, but no difference reached statistical significance. The proportions of *C*. *leptum* group (IV) were significantly lower in patients with both active and inactive IBD compared to healthy controls, whereas the most significant difference was observed in patients with inactive UC (*P* < 0.001). The *F*. *prausnitzii* proportions were also lower in patients with active and inactive IBD than in controls with the most significant difference being observed in patients with active CD (*P* < 0.01), while the difference for cases with active UC did not reach a statistical significance.

**Table 4:** Quantification of bacterial in biopsies and blood microbiota.

	% (mean ± SD) of the bacterial species tested						
Biopsies	*E*. *coli*	*Lactobacillus spp*.	*Bifidobacterium spp*.	*Bacteroides spp*.	*C*. *coccoides group (XIVa)*	*C*. *leptum group (IV)*	*F*. *prausnitzii*
Active CD	10.42 ± 7.92	3.62 ± 2.31	3.78 ± 3.33	21.44 ±18.72	5.53 ± 3.71	4.81 ± 3.60	2.36 ± 1.75
Inactive CD	6.44 ± 2.25	2.45 ± 1.96	4.21 ± 3.98	20.33 ± 18.21	5.12 ± 3.43	3.99 ± 2.82	3.77 ± 2.68
Active UC	10.89 ± 9.75	2.71 ± 2.46	8.32 ± 3.28	19.55 ± 17.48	10.79 ± 3.28	5.68 ± 3.55	4.24 ± 3.81
Inactive UC	4.87 ± 3.83	2.91 ± 1.66	6.98 ± 3.31	18.69 ± 17.33	11.63 ± 4.46	5.00 ± 3.64	5.85 ± 4.23
Healthy	3.57 ± 1.53	2.21 ± 0.98	3.64 ± 2.87	12.22 ± 9.87	12.5 ± 6.21	14.76 ± 5.99	12.28 ± 7.43

In blood samples, from the bacteria tested, only *E*. *coli*, *C*. *coccoides groups* and *C*. *lectum* group were detected in some of the patients of each group and the respective detection rates are shown in Table 5. The detection rate was 50% for *E*. *coli*, 16.67% for *C*. *coccoides* and 16.67% for *C*. *leptum* group in active CD patients, 33.33% for *E*. *coli*, 33.33% for *C*. *coccoides*, and 16.67% for *C*. *leptum* group in both inactive CD, and active UC patients and 28.57% for *E*. *coli*, 21.43% for *C*. *coccoides* and 7.14% for *C*. *leptum* group in inactive UC patients. In controls, the detection rate was 5% for *E*. *coli*, and 5% for *C*. *coccoides group*. There was no significant difference in the detection rate of the identified bacteria among the groups.

**Table 5:** Detection bacterium rate in blood samples.

	Detection rate of the bacterial species tested						
Biopsies	*E*. *coli*	*Lactobacillus spp*.	*Bifidobacterium spp*.	*Bacteroides spp*.	*C*. *coccoides group (XIVa)*	*C*. *leptum group (IV)*	*F*. *prausnitzii*
Active CD	3/6 (50%)	-	-	-	1/6 (16.67%)	1/6 (16.67%)	-
Inactive CD	2/6 (33.33%)	-	-	-	2/6 (33.33%)	1/6 (16.67%)	-
Active UC	2/6 (33.33%)	-	-	-	2/6 (33.33%)	1/6 (16.67%)	-
Inactive UC	4/14 (28.57%)	-	-	-	3/14 (21.43%)	1/14 (7.14%)	-
Healthy	1/20 (5%)	-	-	-	1/20 (5%)	-	-

*NOD2/CARD15* polymorphisms were identified in a total of 9 IBD patients (28.12%) and 2 controls (10%). In particular, 4 of 6 active CD patients, 3 of 6 inactive CD patients, 1 of 14 active UC patients, and 1 of 6 inactive UC patients were carriers of a *NOD2/CARD15* polymorphism. Polymorphism distribution in the overall series of patients was: 3020insC in 6 patients; R702W in 2 patients, and G908R in one patient. Two of 20 healthy controls presented the R702W *NOD2/CARD15* polymorphism. Six of 23 patients with bacterial DNA in blood (26.09%), and 3 of 9 patients without bacterial DNA in blood (33.33%) showed a *NOD2/CARD15* polymorphism. No significant associations between evidences of bacterial translocation and *NOD2/CARD15* were observed in active and inactive CD and UC cases tested.

## Discussion

In this study, we found that dysbiosis in a Greek population is significant among inflammed, non-inflammed and normal intestinal mucosa in healthy controls. We showed that the total concentration of bacterial DNA was higher in active IBD patients, both in both in intestinal tissue and blood, compared to non-active and healthy samples. No differences among inactive CD and UC cases and healthy controls were detected.

Martinez-Medina et al. reported reduced abundance of *F*. *prausnitzii* in ileocolonic mucosal biopsies from patients with CD [27]. Joossens et al. showed a decrease *in F*. *prausnitzii*, *Dialister invisus*, an uncharacterized species of *Clostridium group* XIVa, in *Bifidobacterium* adolescents, in CD patients compared to healthy controls [28]. Machiels K et al. revealed a lower abundance of *F*. *prausnitzii*, and *Roseburia hominis* in UC patients compared to healthy controls [29]. According to our results there was no significant difference in the *Clostridium g*roup XIVa between the IBD patients and the healthy controls. We showed, in agreement with the previous studies, reduced abundance in *F*. *prausnitzii* in active UC and CD and in inactive CD patients compared to healthy controls. The proportion of *F*. *prausnitzii* did not differ between inactive UC patients and healthy subjects.

Interestingly, in a study conducted in Scotland by Hansen et al. demonstrated a significant increase in *F*. *prausnitzi* in pediatric CD patients compared to controls [8]. This result is in contrast with our findings. Although adults and children are genetically similar, there are important distinctions between adult and pediatric inflammatory bowel disease patients. Environmental factors such as gastrointestinal microbial colonization may be etiological factors of relevance to this distinction.

Bibiloni et al. reported an increase in bacteria belonging to phylum *Bacteroidetes* in biopsies from CD patients [12]. Verma et al. showed that *Bacteroidetes* population was decreased in CD, and UC patients compared to healthy controls [30]. Swidsinski et al. found reduced abundance of *C*. *coccoides*, *E*. *rectale* group, and increased proportion of *Bacteroides* in patients with inflammatory bowel disease compared to those who suffered from irritable bowel syndrome [31]. We observed significant increase of *Bacteroides* spp. in inactive CD and UC patients, in active CD compared to healthy controls. No significant difference was observed regarding the *C*. *coccoides* group (XIVa) between the IBD patients and the controls. Various factors may explain the between-study differences: (1) biopsy or stool samples, (2) location, (3) disease activity (quiescent or active disease), (4) age, (5) diet, (6) smoking, (7) sample size of the studies.

Regarding *NOD2/CARD15*, we showed no significant association between bacterial translocation and *NOD2/CARD15* polymorphisms in active and inactive CD and UC cases tested. Our results differ from the study of Gutierrez et al. which reported that *NOD2/CARD15* variants are independent risk factors for bacterial translocation in CD patients [32]. This inconsistency may be due to different sample size of studies, and different populations. Further research in larger and diverse populations is needed to elucidate the relation between bacterial translocation and *NOD2/CARD15* variants.

In our study, all patients received polyethylene glycol as bowel preparation. This might have altered the profile of colonic mucosa, however since the treatment was given to all patients, this parameter should have acted equivalently among groups. Our controls were adults who underwent screening colonoscopy and had a macroscopically normal colonic mucosa.

It remains controversial whether dysbiosis is a consequence or a cause of intestinal inflammation in CD and UC. It is useful to compare the gut microbiota composition of IBD patients with that of their unaffected relatives, in order to provide evidence relevant to this question. Joossens et al. reported dysbiosis in unaffected relatives of CD patients, although it was different from the dysbiosis observed in CD patients [28]. Varela et al. showed a decrease in *F*.*prausnitzi* both in UC patients and in their first-degree relatives [33]. These results suggest that dysbiosis is caused by environmental factors, rather than being a consequence of inflammation.

Recently, Gutierrez et al [34] evaluated the effect of bacterial DNA translocation on relapse in CD patients in remission. According to the study, the presence of bacterial DNA in blood of Crohn’s disease patients is an independent risk factor of CD exacerbation after 6 months. It increases the risk for relapse. The presence of bacterial DNA in blood increased significant the serum levels of the pro-inflammatory cytokines TNF-a, IL-6, IL-12 and IFN-gamma. It would be relevant to perform several studies specifically aimed at evaluating the relationship between the bacterial DNA load and the severity of immune response in IBD patients to prove a causative role for bacterial DNA translocation in relapse.
